# Supplementary material for: Emergent Plants Improve Nitrogen Uptake Rates by Regulating the Activity of Nitrogen Assimilation Enzymes
Source: Plants (Basel). 2025 May 15;14(10):1484. doi: 10.3390/plants14101484 (PMC12114743; doi:10.3390/plants14101484)
Supplement: Supplementary file 1 [file plants-14-01484-s001.zip › plants-3625196-supplementary.pdf]

Supplementary material:

Table S1 Nitrogen uptake amounts in different aquatic plants

Plants	AMA (μg N)		NTA (μg N)		TN (g·kg^{-1})		TM (g)	
	Stems and Leaves	Roots	Stems and Leaves	Roots	Stems and Leaves	Roots	Stems and Leaves	Roots
PH	38.97±9.95a	28.79±7.87a	41.12±13.90a	19.04±8.09a	10.69±0.74a	5.28±0.31a	9.90±0.77b	8.03±0.90a
TY	40.53±7.60a	19.87±6.26b	44.91±15.08a	16.02±6.94ab	10.04±0.85a	4.80±0.38b	11.45±0.91a	7.45±1.11ab
SC	12.17±5.14c	13.46±5.59c	11.17±4.84c	13.70±6.69b	5.41±0.47c	5.35±0.61a	10.30±2.17b	7.00±1.60b
LY	22.82±8.33b	21.21±8.75b	22.57±6.73b	7.91±2.95c	9.09±0.60b	4.83±0.48b	9.88±1.68b	5.77±0.98c

PH, *Phragmites australis*; TY, *Typha orientalis*; SC, *Scirpus validus*; LY, *Lythrum salicaria*.

AMA, The uptake amount of NH_4^+-N; NTA, The uptake amount of NO_3^--N; TN, The total nitrogen content of plant; TM, The plant biomass.

Table S2 Root traits and photosynthetic parameters in different aquatic plants

Plants	AD (mm)	SRL (m·g^{-1})	SRA (cm^2·g^{-1})	RTD (g·cm^{-3})	CHA (mg·L^{-1})	CHB (mg·L^{-1})	CHR	CHT (mg·L^{-1})	Pn (μmol·m^{-2}·s^{-1})
PH	0.69±0.11a	232.54±34.62b	100.01±10.28a	1.56±0.19a	2.09±0.44a	0.55±0.12b	4.03±1.30a	2.64±0.45a	11.90±1.17a
TY	0.65±0.07a	275.21±28.60a	104.10±15.02a	1.05±0.21b	2.05±0.49a	0.60±0.15ab	3.59±1.02a	2.65±0.57a	10.81±0.96b
SC	0.72±0.16a	218.06±71.37b	59.87±15.19b	1.69±0.85a	2.00±0.29a	0.67±0.10a	3.04±0.77a	2.67±0.28a	11.05±1.20b
LY	0.68±0.14a	208.03±46.94b	46.60±7.55c	1.48±0.28a	1.93±0.44a	0.54±0.15b	3.97±1.73a	2.47±0.42a	10.63±1.16b

PH, *Phragmites australis*; TY, *Typha orientalis*; SC, *Scirpus validus*; LY, *Lythrum salicaria*.

AD, The diameter of fine roots; SRL, The specific root length of fine roots; SRA, The specific root surface area of fine roots; RTD, The tissue density of fine roots; CHA, The content of chlorophyll a; CHB, The content of chlorophyll b; CHR, The ratio of Chlorophyll a to Chlorophyll b; CHT, The total chlorophyll content; Pn, The net photosynthetic rate.
